# Analysis of Prevalence and Risk Factors of Contact Sensitization with respect to the Occupational Profiles in a Greek Patient Cohort: A Retrospective Analysis of a Greek Referral Centre and Future Perspectives

**DOI:** 10.1155/2021/6672506

**Published:** 2021-05-06

**Authors:** Anna Tagka, George I. Lambrou, George K. Matsopoulos, Despoina Fytili, Daphne Mirkopoulou, Alexandra Katsarou, Argyro Chatziioannou, Alexandros Stratigos

**Affiliations:** ^1^First Department of Dermatology and Venereology, “Andreas Syggros” Hospital, National and Kapodistrian University of Athens, Medical School, Ionos Dragoumi 5, 11621 Athens, Greece; ^2^First Department of Pediatrics, Choremeio Research Laboratory, National and Kapodistrian University of Athens, Thivon & Levadeias 8, 11527, Goudi, Athens, Greece; ^3^Biomedical Engineering Laboratory, School of Electrical and Computer Engineering, National Technical University of Athens, Heroon Polytechneiou 9, 15771, Zografou, Athens, Greece; ^4^Department of Occupational and Environmental Medicine, “Thriassio” General Hospital, G. Gennimatas Ave., 19600, Elefsis, Athens, Greece; ^5^First Propaedeutic and Internal Medicine Department, University General Hospital of Thessaloniki, AHEPA, St. Kyriakidis 1, 54636 Thessaloniki, Greece

## Abstract

Contact dermatitis is a frequent skin disorder related to environmental and occupational etiological factors, which could potentially affect all age groups, as well as both genders. The current study is aimed at exploring the patterns of contact sensitization with respect to the population's occupational patterns in Greece. A retrospective analysis was performed in a cohort of 1978 patients from 2014 to 2016. Patients were divided into two categories; blue collars (BlC) and white collars (WhC), as well as detailed occupation was considered. Separation was performed on the basis of their profession, i.e., labor workers and handicraftsmen were sorted to the BlC group, while office employees were sorted to the WhC group. The common allergen in all occupational subgroups was nickel sulphate. The three most prevalent allergens in both BlC and WhC were nickel sulphate 5%, fragrance mix (I) 8%, and Balsam of Peru 25%. WhC males were uniquely sensitized to colophony 20% and formaldehyde 2%, and WhC females were uniquely sensitized to 5-chloro-2-methyl-4-isothiazolin-3-one (CMIT) and neomycin sulphate 20%. Sensitization to allergens manifested occupation-specific patterns. Allergic contact dermatitis surveillance is of great importance towards the clinical and systematic understanding of the disease, especially with respect to the patient's occupational profile.

## 1. Introduction

Occupational dermatitis (OD) is defined as “skin, mucous and attachments changes directly or indirectly caused, conditioned, maintained or aggravated in professional activity or work environment” [1]. The factors that can procure contact dermatitis, related to the occupational profile of the patient, vary and can be classified into biological, physical, or chemical. Studies on the subject are scarce, and the real risk factors and the prevalence of the disease are unknown. One of the main obstacles towards the identification of the disease's prevalence is that it is underreported, either due to the fact that it is not immediately recognized as a problem or affected patients not always seek medical consultancy. Although it is not always recognized as a significant pathological condition, it can affect the quality of life and productivity of labor force. Skin-related conditions can cause an increased allocation of resources, either by the employer, employee, and/or the health system as a whole [2]. The European Society of Contact Dermatitis (ESCD) has issued several guidelines for the prevention and diagnosis of OD, yet it is also believed that exposure to allergens and subsequent sensitization can only be regarded through the specific conditions on a country-by-country basis, according to labor, commercial, social, and national habits. For that reason, a baseline of substances has been set as the “standard” termed the “European baseline series” [3]. This series includes several categories of metals, fragrances, preservatives, rubbers, topical therapeutics, and excipients [4]. Further, substances have been proposed, which have been used only on occasion based on the regional habits studies have been performed in. Patch testing manifests great variations between clinics, laboratories, regions, and countries. These observed variations can mainly be attributed to the systematic effects introduced by patient characteristics, differing exposures, patient selection, or methodological differences. The prevalence of contact dermatitis is also studied by the European Surveillance System on Contact Allergies (ESSCA Network), which collects data from different departments and regions, thus, estimating the overall yield concerning to aforementioned baseline series [3, 5]. The pattern of contact sensitization to a series of allergens included in the European baseline series has already been studied for a number of EU countries by the ESSCA Network [3–7].

In the present study, we have attempted to report the pattern of contact sensitization, with respect to the occupational profile of a Greek patient cohort, through patch testing against a large number of allergens inducing contact dermatitis. To the best of our knowledge, this is the first report concerning this topic.

## 2. Materials and Methods

### 2.1. Patients

A total number of 1978 Greek Caucasian patients (619 M/1359F) were admitted in our laboratory during the period between 2014 and 2016 (total of three years). Patients were recruited based on their dermatological profile, whereas all other biometric and anthropometric criteria were kept random. The mean age of all patients was 45.91 ± 18.60 years, where males were 47.31 ± 20.56 years old and females were 45.27 ± 17.60 years old. The identification of contact sensitization included the detection of at least one positive reaction to the chemical panel used in the present study. The retrospective analysis included routine data collected in the Laboratory of Patch Testing, National Referral Centre of Occupational Dermatoses, University Hospital “*Andreas Syggros*”, National and Kapodistrian University of Athens, Medical School [8]. The results refer to consecutive patients in order to avoid bias due to selective testing. Sensitization in all cases was tested with a battery of 28 allergens according to the European baseline series (with addition or omission of allergens due to individual country circumstances) and additional series aiming to identify sensitizations in order to inform the national baseline of allergens. Our patient population sample is summarized in Table 1. Patients have been stratified according to their profession, based on the International Standard Classification of Occupations (ISCO). Patients were initially separated into “Blue Collars” (BlC) indicating those that performed a handicraft and “White Collars” (WhC) indicating those working in an office environment or performing mostly an intellectual profession. Further on, due to the great variety of professions, we have created a shorter list representing the majority of our population. The final occupational list is presented in Supplementary Table 1, which describes the frequencies of the present cohort's occupational profiles.

#### 2.1.1. Inclusion Criteria and Exclusion Criteria

Patients suspected for dermatitis were included in the present study after going through admittance and diagnosis in our department [4]. In case a patient was under treatment including anti-inflammatory medication, cyclosporine, chronic use of corticosteroids, chemotherapeutics, and/or suffered from other chronic dermatopathies was excluded from the present study.

### 2.2. Patch Testing and Clinical Evaluation

The patch testing procedure has been previously described in detail [4]. Patch testing was performed according to the guidelines of the European Society of Contact Dermatitis [5]. The optimal exposure time was maintained at 48 h. The clinical evaluation took place at 48 h, 2-4 days, and 7 days after the first exposure to allergens, as previously suggested [3, 5]. Allergic reaction was evaluated according to the criteria of the International Contact Dermatitis Research Group (ICDRG) and everything else as negative (including irritant reactions) [4].

### 2.3. Clinical Data

Patients admitted in our department, as well as included in the present study, underwent a clinical evaluation, which entailed the collection of routine data. Data included the collection of demographic, clinical data, and patch testing results related to patients suspected with allergic contact dermatitis, as well as occupation. The results were documented to an electronic database. In the case of the patient's repeated admittance, during our study period, only the initial patch test result was considered as previously described [4].

### 2.4. Data Analysis

Patient's characteristics are presented with absolute and relative frequencies (%) [9–11]. Most common allergens by patient characteristics are also presented with absolute and relative frequencies (%) as previously described [4]. Relative frequencies are calculated with respect to the total population (*n* = 1978) as well as with respect to each subpopulation under investigation. Data are available upon reasonable request.

For comparisons between groups, one-way analysis of variance (ANOVA) was performed for the continuous variables, and Chi-square tests were used for the categorical variables. Post hoc comparisons (adjusted with Bonferroni criterion) were also performed when significant differences (*p* < 0.05) of the estimated variables in ANOVA tests were identified. A value of *p* < 0.05 (two-tailed) was set as the level of significance and in the case of post-hoc comparisons a value of *p* < 0.0125 was set as the level of significance.

### 2.5. Ethics Statement

The protocol of our study was approved by the Institutional Scientific Review Board of the University Hospital “*Andreas Syggros*”, National and Kapodistrian University of Athens, Medical School (Protocol. Nr. 2851/2018), and the ethical considerations were fully consistent with the Declaration of Helsinki (1975, review 2000). The data were kept anonymously, and there is no way to track back to the patient's personal data.

## 3. Results and Discussion

### 3.1. Frequencies of the Most Prevalent Allergens with respect to BlC and WhC Population

As aforementioned, we have separated our population in two main occupational categories: blue collars (BlC) and white collars (WhC) (for professions included in each category, please refer to Supplementary Table 1). Frequencies are presented in brackets with respect to the total population as well as with respect to the subpopulation under investigation. For example, the BlC population frequencies are calculated with respect to the total population (*n* = 1978) and with respect to the BlC population (*n* = 615) such as (*f*% total population, *f*% BlC population).

#### 3.1.1. Frequencies of the Most Prevalent Allergens with respect to the BlC Population

From our results, it appeared that the most prevalent allergen was nickel sulphate 5% (7.33%, 23.58%), followed by other allergens in the following descending order: fragrance mix (I) 8% (3.99%, 12.85%), Balsam Of Peru 25% (2.43%, 7.80%), potassium dichromate 0.5% (2.22%, 7.15%), cobalt chloride 1% (2.07%, 6.67%), paraphenylenediamine 1% (2.02%, 6.50%), ethylenediamine 1% (1.57%, 5.04%), thiomersal 0.1% (1.42%, 4.55%), thiuram mix 1% (1.31%, 4.23%), budesonide 0.01% (0.86%, 2.76%), formaldehyde 2% (0.76%, 2.44%), colophony 20% (0.71%, 2.28%), CMIT (0.66%, 2.11%), Neomycin sulphate 20% (0.66%, 2.11%), and wool alcohols 30% (0.46%, 1.46%) (Table 2).

#### 3.1.2. Frequencies of the Most Prevalent Allergens with respect to the Male BlC Population

In the case of male BlC, it appeared that the most prevalent allergen was potassium dichromate 0.5% (1.26%, 16.03%), nickel sulphate 5% (1.06%, 13.46%), cobalt chloride 1% (1.01%, 12.82%), Balsam of Peru 25% (0.81%, 10.26%), thiomersal 0.1% (0.66%, 8.33%), fragrance mix (I) 8% (0.66%, 8.33%), thiuram mix 1% (0.66%, 8.33%), ethylenediamine 1% (0.61%, 7.69%), paraphenylenediamine 1% (0.56%, 7.05%), budesonide 0.01% (0.30%, 3.85%), epoxy resin 1% (0.30%, 3.85%), CMIT (0.25%, 3.21%), formaldehyde 2% (0.25%, 3.21%), wool alcohols 30% (0.20%, 2.56%), and benzocaine 5% (0.20%, 2.56%) (Table 2).

#### 3.1.3. Frequencies of the Most Prevalent Allergens with respect to the Female BlC Population

Similarly, for the female BlC, it appeared that the most prevalent allergen was nickel sulphate 5% (6.27%, 27.02%), fragrance mix (I) 8% (3.34%, 14.38%), Balsam of Peru 25% (1.62%, 6.97%), paraphenylenediamine 1% (1.47%, 6.32%), cobalt chloride 1% (1.06%, 4.58%), ethylenediamine 1% (0.96%, 4.14%), potassium dichromate 0.5% (0.96%, 4.14%), thiomersal 0.1% (0.76%, 3.27%), thiuram mix 1% (0.66%, 2.83%), budesonide 0.01% (0.56%, 2.40%), colophony 20% (0.56%, 2.40%), formaldehyde 2% (0.51%, 2.18%), Neomycin sulphate 20% (0.51%, 2.18%), CMIT (0.40%, 1.74%), quaternium 15 1% (0.30%, 1.31%), paratertiary butyl phenol 1% (0.30%, 1.31%), and wool alcohols 30% (0.25%, 1.09%) (Table 2).

#### 3.1.4. Frequencies of the Most Prevalent Allergens with respect to the WhC Population

Frequencies are presented in brackets with respect to the total population as well as with respect to the subpopulation under investigation. Thus, in the present case, frequencies are calculated with respect to the total population (*n* = 1978) and with respect to the WhC population (*n* = 1363) such as (*f*% total population and *f*% WhC population). From our results, it appeared that the most prevalent allergen was nickel sulphate 5% (18.55%, 26.93%), fragrance mix (I) 8% (10.21%, 14.82%), Balsam of Peru 25% (7.33%, 10.64%), thiomersal 0.1% (5.71%, 8.29%), cobalt chloride 1% (5.46%, 7.92%), ethylenediamine 1% (3.54%, 5.14%), potassium dichromate 0.5% (3.39%, 4.92%), paraphenylenediamine 1% (2.88%, 4.18%), neomycin sulphate 20% (2.22%, 3.23%), CMIT (2.17%, 3.15%), formaldehyde 2% (2.17%, 3.15%), budesonide 0.01% (1.92%, 2.79%), colophony 20% (1.37%, 1.98%), black rubber mix 0.1% (1.21%, 1.76%), wool alcohols 30% (1.06%, 1.54%), thiuram mix 1% (1.06%, 1.54%), paratertiary butyl phenol 1% (0.96%, 1.39%), paraben mix 15% (0.91%, 1.32%), quaternium 15 1% (0.71%, 1.03%), benzocaine 5% (0.66%, 0.95%), primin 0.01% (0.56%, 0.81%), mercapto mix 2% (0.51%, 0.73%), mercury 0.05% (0.35%, 0.51%), quinoline mix 6% (0.35%, 0.51%), benzalkonium chloride 0.1% (0.25%, 0.37%), epoxy resin 1% (0.25%, 0.37%), and MBT 2% (0.20%, 0.29%) (Table 3).

#### 3.1.5. Frequencies of the Most Prevalent Allergens with respect to the Male WhC Population

Frequencies are presented in brackets with respect to the total population as well as with respect to the subpopulation under investigation. Thus, in the present case, frequencies are calculated with respect to the total population (*n* = 1978) and with respect to the male WhC population (*n* = 463) such as (*f*% total population and *f*% male WhC population). From our results, it appeared that the most prevalent allergen was Balsam of Peru 25% (3.13%, 13.39%), fragrance mix (I) 8% (2.98%, 12.74%), nickel sulphate 5% (2.38%, 10.15%), thiomersal 0.1% (1.77%, 7.56%), ethylenediamine 1% (1.67%, 7.13%), potassium dichromate 0.5% (1.26%, 5.40%), cobalt chloride 1% (1.21%, 5.18%), budesonide 0.01% (0.91%, 3.89%), colophony 20% (0.71%, 3.02%), formaldehyde 2% (0.66%, 2.81%), wool alcohols 30% (0.46%, 1.94%), Neomycin sulphate 20% (0.46%, 1.94%), CMIT (0.35%, 1.51%), paratertiary butyl phenol 1% (0.35%, 1.51%), paraben mix 15% (0.35%, 1.51%), thiuram mix 1% (0.35%, 1.51%), paraphenylenediamine 1% (0.30%, 1.30%), primin 0.01% (0.25%, 1.08%), black rubber mix 0.1% (0.20%, 0.86%), quaternium 15 1% (0.15%, 0.65%), MBT 2% (0.10%, 0.43%), mercury 0.05% (0.10%, 0.43%), epoxy resin 1% (0.10%, 0.43%), benzocaine 5% (0.10%, 0.43%), benzalkonium chloride 0.1% (0.05%, 0.22%), and quinoline mix 6% (0.05%, 0.22%) (Table 3).

#### 3.1.6. Frequencies of the Most Prevalent Allergens with respect to the Female WhC Population

Frequencies are presented in brackets with respect to the total population as well as with respect to the subpopulation under investigation. Thus, in the present case, frequencies are calculated with respect to the total population (*n* = 1978) and with respect to the female WhC population (*n* = 900) such as (*f*% total population and *f*% female WhC population). From our results, it appeared that the most prevalent allergen was nickel sulphate 5% (16.18%, 35.56%), fragrance mix (I) 8% (7.23%, 15.89%), cobalt chloride 1% (4.25%, 9.33%), Balsam of Peru 25% (4.20%, 9.22%), thiomersal 0.1% (3.94%, 8.67%), paraphenylenediamine 1% (2.58%, 5.67%), potassium dichromate 0.5% (2.12%, 4.67%), ethylenediamine 1% (1.87%, 4.11%), CMIT (1.82%, 4.00%), Neomycin sulphate 20% (1.77%, 3.89%), formaldehyde 2% (1.52%, 3.33%), budesonide 0.01% (1.01%, 2.22%), black rubber mix 0.1% (1.01%, 2.22%), thiuram mix 1% (0.71%, 1.56%), colophony 20% (0.66%, 1.44%), paratertiary butyl phenol 1% (0.61%, 1.33%), wool alcohols 30% (0.61%, 1.33%), quaternium 15 1% (0.56%, 1.22%), paraben mix 15% (0.56%, 1.22%), benzocaine 5% (0.56%, 1.22%), mercapto mix 2% (0.51%, 1.11%), primin 0.01% (0.30%, 0.67%), quinoline mix 6% (0.30%, 0.67%), mercury 0.05% (0.25%, 0.56%), benzalkonium chloride 0.1% (0.20%, 0.44%), epoxy resin 1% (0.15%, 0.33%), and MBT 2% (0.10%, 0.22%) (Table 3).

### 3.2. Common Allergens between BlC and WhC

If we examine Tables 2 and 3 more closely, we observe that several allergens are unique for each subpopulation cohort. In particular, it appeared that seven allergens are common to all subgroups, while allergens were found to be uniquely represented in each subpopulation. Such allergens were (a) WhC females were uniquely positive to CMIT and neomycin sulphate 20%, (b) WhC males were uniquely positive to colophony 20% and formaldehyde 2%, (c) thiuram mix 1% was uniquely positive in BlC males and BlC females, and (d) paraphenylenediamine 1% was uniquely positive in BlC females, BlC males WhC females.

### 3.3. Differences in Total Positive Allergens with respect to Gender and Occupation

**Figure 1:**
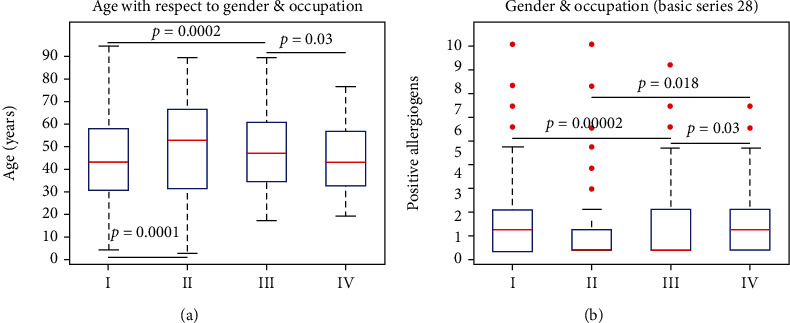
Age and number of total positive allergens with respect to gender and occupation. Significant differences were observed between female WhC (*p* = 0.0001) and male WhC (a). Also, a significant difference was observed between female WhC (*p* = 0.0002) and female BlC (a). Further on, we have estimated the total number of allergens in each subgroup. In that case, a significant difference was observed between female WhC and female BlC (*p* ≤ 0.001) (b). (Legend: WhC: white collars; BlC: blue collars; I: female white collars; II: male white collars; III: female blue collars; IV: male blue collars).

In the present analysis, we have estimated the total positive allergens in each subgroup, thus, with respect to gender and occupation. Total allergens were estimated by counting the allergens in which each patient manifested a positive test and then these data were compared with respect to gender and occupation. Hence, significant differences were observed between female WhC and male WhC (*p* ≤ 0.001) (Figure 1(a)), with respect to their age, with male WhC being older than all other groups, as well as between female WhC and female BlC (*p* = 0.0002) (Figure 1(a)). Further on, the estimated total number of allergens in each subgroup manifested significant differences between female WhC and female BlC (*p* ≤ 0.001) (Figure 1(b)). This was an interesting finding since it would be expected for the BlC subgroup to manifest a higher frequency of allergens per patient due to the expected higher corporal contact of allergens, yet WhC manifested a higher frequency of positive allergens per patient as compared to BlC.

### 3.4. Risk Assessment of Allergens with respect to Occupation

Risk measures, with respect to occupation, manifested several interesting results. In particular, occupational dermatitis showed that the BlC group was 80% more likely to have preexisting occupational exposure (OE) than the WhC group (OR = 4.91, *p* ~ 0). A similar result was obtained for the presence of upper extremity dermatitis (HD), where BlC were 33.3% more likely to have preexisting upper extremity dermatitis than WhC (OR = 1.72, *p* ~ 0) and familial history of dermatitis (FHist) (OR = 1.52, *p* = 0.0017). On the contrary, WhC appeared to be 8% more likely to have facial dermatitis (FD) than BlC (OR = 0.77, *p* = 0.014), 10% more likely to have a preexisting lower extremity dermatitis (LD) (OR = 0.65, *p* = 0.0002), 11% more likely to have preexisting trunk dermatitis (TD) (OR = 0.67, *p* = 0.0003), and finally, 5% more likely to have more than two concurrent dermatitis sites (OR = 0.75, *p* = 0.026). In the case of positive reactions to allergens, the BlC group was 3% more likely to be positive in thiuram mix 1%, less likely (0.1%) to be positive in epoxy resin 1%, 16% more likely to be positive in thiuram mix, abiet acid 10%, and 8% more likely to be positive for ammonium persulfate 2.5% than the WhC group. On the contrary, the BlC group was 4% less likely to be positive for Balsam of Peru 25%, 4% less likely to be positive for thiomersal 0.1%, 15% less likely to be positive for D. pteronyssimus, and 11% less likely for a positive reaction for D. farinae. Results are also summarized in Table 4.

### 3.5. Frequencies of the Most Prevalent Allergens with respect to the Detailed Occupation

Finally, in Supplementary Table 2, the frequencies of the most prevalent allergens in the detailed occupations are presented. We have also used an algorithmic approach in order to identify possible common allergens among all patient occupational subgroups. In the case of BlC, WhC nickel sulphate 5% was found to be the common allergen in all patients, with a mean frequency of 0.91% and 0.62%, respectively.

## 4. Discussion

The present study has attempted to report and analyze the prevalence of contact sensitization in a Greek patient cohort with respect to the occupational status of our patient cohort. We have separated our patient cohort in two main categories, based on their occupational status, which was BlC and WhC. Our results manifested that contact sensitization was prevalent in both occupational subgroups as well as with respect to gender and occupational group. The investigation of sensitization profiles based on the occupational profiles of a patient cohort can possibly assist towards the identification of the possible sources of sensitization in terms of habitual circumstances or lifestyles. For example, in our study, the anticipated sensitization to fragrance mix was due to the use of cosmetics, as it appeared that it was more frequent to white collars [12]. On the other hand, Balsam of Peru (*Myroxylon pereirae* resin) sensitized our population also due to cosmetic products [13]. Colophony sensitization in our patient cohort was not clear, yet the highest incidence was observed in retirees (Supplementary Table 2), who use such products made of resin (e.g., backgammon and worry beads) [14, 15]. Another interesting example is derived from formaldehyde, whose indoor exposure is a major health concern, especially for school-age children who spend most of their time indoors [16, 17]. This was in agreement with our results since sensitization was more frequent to professions that stay mostly indoors such as retirees, clerks, students, and teachers (Supplementary Table 2).

In the present work, we have found that nickel sulphate 5% is the most prevalent allergen in all occupational subgroups, the general population, which is in agreement with previous reports [18, 19]. In a recent study, it has been reported that nickel sulphate prevalence of contact dermatitis ranges between 8% to 19% in adults and 8% to 10% in children [20]. Other studies have reported that contact dermatitis from nickel sulphate ranged from 11.9% in Denmark to 26.4% in Spain [18, 19, 21]. Thus, our results for nickel sulphate sensitization were also in agreement to the findings in South European countries. Interestingly, we have shown that WhC were uniquely sensitized to CMIT and neomycin sulphate. Previous reports have shown that neomycin sulphate is the most common allergen in medication responsible dermatitis [22]. On the other hand, male WhC were uniquely sensitized in colophony and formaldehyde. The prevalence of sensitization in the total population was calculated to be 2.02% [23], while male WhC prevalence was 0.71%, much lower than that reported from other studies and in particular 4.8% [24]. Also, thiuram mix 1% was uniquely positive in BlC males and BlC females which is in agreement with previous studies for workers (BlC) [7, 25, 26]. Finally, paraphenylenediamine 1% was uniquely positive in BlC females, BlC males, and WhC females. Our result is in agreement with previous studies that report sensitization to paraphenylenediamine as occupational-related dermatitis [27–29]. Our data suggest that the specialized clinician could take into account the occupational profile of the patient, in order to better assess the contact sensitization and underlying factors that might have led to the allergic reactions.

Another interesting finding was that WhC and BlC manifested a reversed pattern with respect to age. In particular, male WhC patients were older as compared to females WhC, and the opposite was manifested for BlC, where female BlC were older as compared to male BlC. To the best of our knowledge, there are no previous reports for the Greek population. In addition, another interesting finding was the fact that female WhC manifested significantly higher positive allergens as compared to male WhC and female BlC, yet the same as compared to male BlC. It would be expected that BlC due to their occupational profiles should manifest more positive allergens as compared to WhC. This result indicates that sensitization includes more factors than the mere immunological response to allergens. To the best of our knowledge, there are no previous reports on this finding.

Further on, the finding that the BlC group was 80% more likely to have preexisting occupational exposure (OE) as compared to the WhC group agreed with the separation of our cohort between “hand workers” and “clerks.” Similarly, BlC were more prone to upper extremities preexisting dermatitis, supporting the classification of our cohort, also indicating that the occupational profile of a patient cohort could prove useful towards the prediction and prevention of allergens. This was also supported by the finding that WhC were more prone to preexisting facial dermatitis as compared to BlC. In addition, the identification of unique “sensitizers” for each of the occupational groups could prove useful for the prevention of contact dermatitis, besides the already known most prevalent allergens. In many cases, sensitization takes place not only due to exposure in one allergen but due to exposure to multiple allergens. It is possible that our approach could prove useful towards the detection of multiple exposures to allergens [30].

Surveillance of contact dermatitis has proven a useful tool since it is essential to unravel time trends in allergic pathologies or in order to discover patterns of lifestyle, environmental stimuli, and occupational hazards [6]. Further on, such surveillance programs are essential for public health policy making and thus the establishments of preventive policies with respect to dermatological diseases. This aspect, i.e., of public health prevention and policy making, is also linked to quality of life, which is not thoroughly investigated in allergic dermatitis patients [2, 31]. In that sense, epidemiological studies are important, meaning that it is crucial to have data available for the quality of life of allergic contact dermatitis patients.

Recent reports have highlighted both the significance as well as the importance of contact dermatitis studies, which is reinforced by the fact that a European surveillance report is published by collecting data from several European countries and comparing contact dermatitis in a country- and ethnic-dependent manner. In a previous study, we have reported the calculation of an extended MOAHLFA index, a very important parameter in the evaluation of allergic contact dermatitis [23]. Our studies were in agreement with other recent reports that have calculated this index of other European countries. In particular, in Table 5, we present a comparative study of the MOAHLFA index in several European countries as well as our present study.

## 5. Study Limitations

One of the study limitations is the possible first-stage selection bias, which cannot be ruled out. One further difficulty is the comparison of occupational contact sensitization prevalence between countries as well as the inherent differences between the similar departments among different countries. Further on, it would be extremely useful for the understanding of contact sensitization dynamics to be able to perform time-dependent studies, thus, to be able to find patterns of sensitizations and also predict future trends.

## 6. Future Perspectives

In the present study, we have investigated the epidemiological aspects of contact dermatitis by taking into account the occupational profiles of our cohort. This type of analysis will continue by expanding the investigated population, but also we will use improved technological tools for the evaluation and analysis of contact dermatitis. The present cohort under investigation was examined clinically and diagnosis was given by applying classical clinical methods, i.e., the physicians and health professionals' experience and perception. This procedure will be amended by the use of new computational tools, including artificial intelligence (AI), machine learning, and algorithmic approaches in an attempt to produce a more automated flow for the diagnosis of contact dermatitis.

## 7. Conclusions

The present study showed that the prevalence of contact sensitization in all populations, irrespectively of occupational profile, was highest for nickel. Accordingly, the five most prevalent allergens in BlC were nickel sulphate 5%, fragrance mix (i) 8%, Balsam of Peru 25%, potassium dichromate 0.5%, and cobalt chloride 1%. Similarly, the five most prevalent allergens in WhC were nickel sulphate 5%, fragrance mix (i) 8%, Balsam of Peru 25%, thiomersal 0.1%, and cobalt chloride 1%. WhC males were uniquely sensitized to colophony 20% and formaldehyde 2%, and WhC females were uniquely sensitized to CMIT and neomycin sulphate 20%. Our findings indicated that patient stratification based on their occupational profiles could prove useful for their detection of allergen cross-reactions as well as prevention of allergic sensitization due to the occupational profile.

The biological mechanisms behind contact dermatitis are largely unknown. As contact dermatitis is influenced by environmental as well as genetic factors, epidemiological studies are considered of crucial importance towards the understanding of the condition and the establishment of further effective clinical and laboratory tests.

## Figures and Tables

**Table 1:** Patient population and age (^¥^*p* value indicates differences between male and female population) (reproduced from Tagka et al. (2020) [4]).

Population				
	Males (*n*)	Females (*n*)	Sum	*p* value^¥^
2014	211	457	668	
2015	201	453	654	
2016	207	449	656	
Sum	619	1359	1978	

Age (years) (mean ± st deviation)				
	Males	Females	Both genders	*p* value^¥^
2014	45.49 ± 21.17	44.66 ± 17.85	44.92 ± 18.95	0.59
2015	49.35 ± 20.56	44.53 ± 17.26	46.01 ± 18.46	0.002
2016	47.18 ± 19.85	46.64 ± 17.67	46.81 ± 18.37	0.73
All years	47.31 ± 20.56	45.27 ± 17.61	45.91 ± 18.60	0.02

**Table 2:** Frequencies of the top-15 allergens of the EBS. Frequencies are presented in descending order calculated with respect to the BlC population (*n* = 615), for the total population as well as with respect to gender.

	Blue collars (*n* = 615)								
	All blue collars			Males (*n* = 156)			Females (*n* = 459)		
	Absolute frequency	*f*(%) total population	*f*(%) blue collar population	Absolute frequency	*f*(%) total population	*f*(%) male blue collar population	Absolute frequency	*f*(%) total population	*f*(%) female blue collar population
Nickel sulphate 5%	145	7.33%	23.58%	21	1.06%	13.46%	124	6.27%	27.02%
Fragrance mix (I) 8%	79	3.99%	12.85%	13	0.66%	8.33%	66	3.34%	14.38%
Balsam of Peru 25%	48	2.43%	7.80%	16	0.81%	10.26%	32	1.62%	6.97%
Potassium dichromate 0.5%	44	2.22%	7.15%	25	1.26%	16.03%	19	0.96%	4.14%
Cobalt chloride 1%	41	2.07%	6.67%	20	1.01%	12.82%	21	1.06%	4.58%
Paraphenylenediamine 1%	40	2.02%	6.50%	11	0.56%	7.05%	29	1.47%	6.32%
Ethylenediamine 1%	31	1.57%	5.04%	12	0.61%	7.69%	19	0.96%	4.14%
Thiomersal 0.1%	28	1.42%	4.55%	13	0.66%	8.33%	15	0.76%	3.27%
Thiuram mix 1%	26	1.31%	4.23%	13	0.66%	8.33%	13	0.66%	2.83%
Budesonide 0.01%	17	0.86%	2.76%	6	0.30%	3.85%	11	0.56%	2.40%
Formaldehyde 2%	15	0.76%	2.44%	5	0.25%	3.21%	10	0.51%	2.18%
Colophony 20%	14	0.71%	2.28%	3	0.15%	1.92%	11	0.56%	2.40%
CMIT	13	0.66%	2.11%	5	0.25%	3.21%	8	0.40%	1.74%
Neomycin sulphate 20%	13	0.66%	2.11%	3	0.15%	1.92%	10	0.51%	2.18%
Wool alcohols 30%	9	0.46%	1.46%	4	0.20%	2.56%	5	0.25%	1.09%

**Table 3:** Frequencies of the top-15 allergens of the EBS. Frequencies are presented in descending order calculated with respect to the WhC population (*n* = 1363), for the total population as well as with respect to gender.

	White collars (*n* = 1363)								
	All white collars			Males (*n* = 463)			Females (*n* = 900)		
	Absolute frequency	*f*(%) total population	*f*(%) white collar population	Absolute frequency	*f*(%) total population	*f*(%) male white collar population	Absolute frequency	*f*(%) total population	*f*(%) female white collar population
Nickel sulphate 5%	367	18.55%	26.93%	47	2.38%	10.15%	320	16.18%	35.56%
Fragrance mix (I) 8%	202	10.21%	14.82%	59	2.98%	12.74%	143	7.23%	15.89%
Balsam of Peru 25%	145	7.33%	10.64%	62	3.13%	13.39%	83	4.20%	9.22%
Thiomersal 0.1%	113	5.71%	8.29%	35	1.77%	7.56%	78	3.94%	8.67%
Cobalt chloride 1%	108	5.46%	7.92%	24	1.21%	5.18%	84	4.25%	9.33%
Ethylenediamine 1%	70	3.54%	5.14%	33	1.67%	7.13%	37	1.87%	4.11%
Potassium dichromate 0.5%	67	3.39%	4.92%	25	1.26%	5.40%	42	2.12%	4.67%
Paraphenylenediamine 1%	57	2.88%	4.18%	6	0.30%	1.30%	51	2.58%	5.67%
Neomycin sulphate 20%	44	2.22%	3.23%	9	0.46%	1.94%	35	1.77%	3.89%
CMIT	43	2.17%	3.15%	7	0.35%	1.51%	36	1.82%	4.00%
Formaldehyde 2%	43	2.17%	3.15%	13	0.66%	2.81%	30	1.52%	3.33%
Budesonide 0.01%	38	1.92%	2.79%	18	0.91%	3.89%	20	1.01%	2.22%
Colophony 20%	27	1.37%	1.98%	14	0.71%	3.02%	13	0.66%	1.44%
Black rubber mix 0.1%	24	1.21%	1.76%	4	0.20%	0.86%	20	1.01%	2.22%
Wool alcohols 30%	21	1.06%	1.54%	9	0.46%	1.94%	12	0.61%	1.33%

**Table 4:** Risk ratios of patients with respect to their occupation, as compared to other dermatological factors (Legend: OD: occupational dermatitis; HD: hand dermatitis; FD: lower extremities dermatitis; TD: trunk dermatitis, >2 SITES: patients that manifested more than two concurrent positive sites of dermatitis; Fhist: familial history of atopy; OR: odds ratio; RR: relative risk; AR: absolute risk).

			Odds ratio (OR)	Fishers test *p* value
Occupational dermatitis	OD_NO	OD_YES	4.9098	*p* ≤ 0.001
White collar	1083	280		
Blue collar	271	344		
Hand dermatitis	HD_NO	HD_YES	1.7254	*p* ≤ 0.001
White collar	736	627		
Blue collar	249	366		
Face dermatitis	FD_NO	FD_YES	0.7686	0.0141
White collar	908	455		
Blue collar	444	171		
Leg dermatitis	LD_NO	LD_YES	0.6448	0.0002
White collar	999	364		
Blue collar	498	117		
Trunk dermatitis	TD_NO	TD_YES	0.6705	0.0003
White collar	915	448		
Blue collar	463	152		
Dermatitis in more than 2 sites	>2_SITES_NO	>2_SITES_YES	0.7488	0.0259
White collar	1085	278		
Blue collar	516	99		
Familial history	Fhist_YES	Fhist_NO	1.5206	0.0017
White collar	276	1087		
Blue collar	88	527		
Thiuram mix 1%	Negative	Positive	2.8209	0.0006
White collar	1342	21		
Blue collar	589	26		
Balsam of Peru 25%	Negative	Positive	0.7111	0.0498
White collar	1218	145		
Blue collar	567	48		
Epoxy resin 1%	Negative	Positive	3.5796	0.0301
White collar	1358	5		
Blue collar	607	8		
Thiomersal 0.1%	Negative	Positive	0.5277	0.0024
White collar	1250	113		
Blue collar	587	28		
Abiet acid 10%	Negative	Positive	6.5463	0.0001
White collar	202	6		
Blue collar	72	14		
Ammonium persulfate 2.5%	Negative	Positive	7.0085	0.0030
White collar	205	3		
Blue collar	78	8		
D. pteronyssinus	Negative	Positive	0.2593	0.0039
White collar	168	40		
Blue collar	81	5		
D. farinae	Negative	Positive	0.2785	0.0162
White collar	177	31		
Blue collar	82	4		

**Table 5:** Comparative table of the MOAHLFA index with respect to countries, including the present study (adopted from Uter et al. and Tagka et al. (2015, 2019) [19, 23]).

Country/department	N (test)	M	O	A	H	L	F	A
AT/Graz	1113	26.1	17	20	26.2	6.8	16.7	63.9
CH total	4990	38.9	13.8	19	26.2	7	16.3	65.8
DE total	7628	40.4	32	24.8	40.9	7.1	11	70.3
DK/Gentofte/Copenhagen	2582	30.4	21.9	18.2	38.8	1.8	27.1	64.9
ES total	4257	31.7	12	13.8	26.4	7.8	13.7	65
FI total	1057	37.9	44.2	30.6	59.8	3.2	4.4	56.6
IT total	9267	33.4	4.6	16.6	21.6	7.3	12.3	51.2
LT/Kaunas	865	19.5	13.3	11.3	25.1	11.2	22.9	63.7
NL total	4385	34	18.4	34.5	20.5	4.5	19.4	56.8
PL total	2828	28.9	22.4	13.2	29.2	3.9	15.1	56.2
SI total	5224	30.7	—	—	—	—	—	58.8
UK total	15 532	31.9	9.7	34.6	28	6.3	27.5	58.2
EL total (Tagka et al. (2019))	1978	31.29	31.55	34.98	50.20	24.31	31.65	58.29

## Data Availability

The datasets used and/or analyzed during the current study are available from the corresponding author on reasonable request.

## Supplementary Materials

Supplementary Materials Patients have been stratified according to their profession, based on the International Standard Classification of Occupations (ISCO). Due to the great variety of professions, we have created a shorter list representing the majority of our population. This final occupational list of the patient cohort is summarized in Supplementary Table 1, which also summarizes the frequencies of the present cohort's occupational profiles. In Supplementary Table 2, we have used the occupational taxonomy of Supplementary Table 1 and presented the frequencies of the most prevalent allergens in each of these occupations.
