# Supplementary material for: Analysis of Prevalence and Risk Factors of Contact Sensitization with respect to the Occupational Profiles in a Greek Patient Cohort: A Retrospective Analysis of a Greek Referral Centre and Future Perspectives
Source: Biomed Res Int. 2021 May 6;2021:6672506. doi: 10.1155/2021/6672506 (PMC8121586; doi:10.1155/2021/6672506)
Supplement: Supplementary Materials — Patients have been stratified according to their profession, based on the International Standard Classification of Occupations (ISCO). Due to the great variety of professions, we have created a shorter list representing the majority of our population. This final occupational list of the patient cohort is summarized in Supplementary Table 1, which also summarizes the frequencies of the present cohort's occupational profiles. In Supplementary Table 2, we have used the occupational taxonomy of Supplementary Table 1 and presented the frequencies of the most prevalent allergens in each of these occupations. [file 6672506.f1.zip › 6672506.f1.docx]

| **Inv.** | **OCCUPATION** | | ***n*** |
| --- | --- | --- | --- |
|  | **BLUE COLLARS** | | ***621*** |
| **1.** | **AGRICULTURE** | BLUE COLLAR | **27** |
| **6.** | **BLUE COLLAR** | BLUE COLLAR | **159** |
| **10.** | **COOK** | BLUE COLLAR | **15** |
| **11.** | **COSMETICS** | BLUE COLLAR | **121** |
| **13.** | **DOMESTIC** | BLUE COLLAR | **239** |
| **14.** | **DOMESTICS** | BLUE COLLAR | **38** |
| **20.** | **NAVY** | BLUE COLLAR | **7** |
| **38.** | **WAITER** | BLUE COLLAR | **15** |
|  | **WHITE COLLARS** | | ***1266*** |
| **2.** | **ARMY OFFICER** | WHITE COLLAR | **4** |
| **3.** | **ARTIST** | WHITE COLLAR | **29** |
| **4.** | **ATHLETE** | WHITE COLLAR | **12** |
| **5.** | **ATTORNEY** | WHITE COLLAR | **23** |
| **7.** | **BUSINESSMAN** | WHITE COLLAR | **43** |
| **8.** | **CHILD** | WHITE COLLAR | **6** |
| **9..** | **CLERK** | WHITE COLLAR | **243** |
| **12.** | **DENTIST** | WHITE COLLAR | **6** |
| **15.** | **DRIVER** | WHITE COLLAR | **24** |
| **16.** | **ECONOMICS** | WHITE COLLAR | **30** |
| **17.** | **ENGINEER** | WHITE COLLAR | **43** |
| **18.** | **INFORMATICS** | WHITE COLLAR | **6** |
| **19.** | **JOURNALIST** | WHITE COLLAR | **5** |
| **21.** | **NURSE** | WHITE COLLAR | **36** |
| **22.** | **OTHER** | WHITE COLLAR | **3** |
| **23.** | **PARAMEDICAL** | WHITE COLLAR | **25** |
| **24.** | **PHARMACIST** | WHITE COLLAR | **4** |
| **25.** | **PHYSICIAN** | WHITE COLLAR | **18** |
| **26.** | **PROFESSOR** | WHITE COLLAR | **4** |
| **27..** | **PSYCHOLOGY** | WHITE COLLAR | **8** |
| **28.** | **PUBLIC SERVANTS** | WHITE COLLAR | **33** |
| **29.** | **PUPIL** | WHITE COLLAR | **109** |
| **30.** | **RETIRED** | WHITE COLLAR | **352** |
| **31.** | **SCIENTIST** | WHITE COLLAR | **16** |
| **32.** | **SECURITY OFFICER** | WHITE COLLAR | **13** |
| **33.** | **SOCIOLOGY** | WHITE COLLAR | **7** |
| **34..** | **STUDENT** | WHITE COLLAR | **86** |
| **35.** | **TEACHER** | WHITE COLLAR | **78** |
| **36.** | **UNEMPLOYED** | WHITE COLLAR | **89** |
| **37.** | **VETERINERIAN** | WHITE COLLAR | **2** |

**Supplementary Table 1**. Occupations evaluated in the present study, in alphabetical order.
