# Supplementary material for: Analysis of Prevalence and Risk Factors of Contact Sensitization with respect to the Occupational Profiles in a Greek Patient Cohort: A Retrospective Analysis of a Greek Referral Centre and Future Perspectives
Source: Biomed Res Int. 2021 May 6;2021:6672506. doi: 10.1155/2021/6672506 (PMC8121586; doi:10.1155/2021/6672506)
Supplement: Supplementary Materials — Patients have been stratified according to their profession, based on the International Standard Classification of Occupations (ISCO). Due to the great variety of professions, we have created a shorter list representing the majority of our population. This final occupational list of the patient cohort is summarized in Supplementary Table 1, which also summarizes the frequencies of the present cohort's occupational profiles. In Supplementary Table 2, we have used the occupational taxonomy of Supplementary Table 1 and presented the frequencies of the most prevalent allergens in each of these occupations. [file 6672506.f1.zip › 6672506.f2.docx]

| **ECONOMICS (WC, *n*=30)** | | | |
| --- | --- | --- | --- |
|  | **Absolute *f(n)*** | ***f(%)* Total Population** | ***f(%)* Economics Population** |
| **NICKEL SULPHATE 5%** | 10 | 0.51% | 33.33% |
| **THIOMERSAL 0.1%** | 4 | 0.20% | 13.33% |
| **FRAGRANCE MIX (I) 8%** | 3 | 0.15% | 10.00% |
| **BUDESONIDE 0.01%** | 2 | 0.10% | 6.67% |
| **5-CHLORO-2-METHYL-4-ITZ-3** | 1 | 0.05% | 3.33% |
| **WOOL ALCOHOLS 30%** | 1 | 0.05% | 3.33% |
| **BLACK RUBBER MIX 0.1%** | 1 | 0.05% | 3.33% |
| **BALSAM OF PERU 25%** | 1 | 0.05% | 3.33% |
| **FORMALDEHYDE 2%** | 1 | 0.05% | 3.33% |
| **PARAPHENYLENEDIAMINE 1%** | 1 | 0.05% | 3.33% |
| **NEOMYCIN SULPHATE 20%** | 1 | 0.05% | 3.33% |
| **POTASSIUM DICHROMATE 0.5%** | 1 | 0.05% | 3.33% |
| **RETIRED (WC, *n*=352 )** | | | |
|  | **Absolute *f(n)*** | ***f(%)* Total Population** | ***f(%)* Retired Population** |
| **BALSAM OF PERU 25%** | 66 | 3.34% | 18.75% |
| **FRAGRANCE MIX (I) 8%** | 64 | 3.24% | 18.18% |
| **NICKEL SULPHATE 5%** | 52 | 2.63% | 14.77% |
| **ETHYLENEDIAMINE 1%** | 22 | 1.11% | 6.25% |
| **POTASSIUM DICHROMATE 0.5%** | 20 | 1.01% | 5.68% |
| **BUDESONIDE 0.01%** | 15 | 0.76% | 4.26% |
| **COBALT CHLORIDE 1%** | 15 | 0.76% | 4.26% |
| **FORMALDEHYDE 2%** | 13 | 0.66% | 3.69% |
| **NEOMYCIN SULPHATE 20%** | 11 | 0.56% | 3.13% |
| **COLOPHONY 20%** | 10 | 0.51% | 2.84% |
| **THIURAM MIX 1%** | 7 | 0.35% | 1.99% |
| **PARAPHENYLENEDIAMINE 1%** | 6 | 0.30% | 1.70% |
| **5-CHLORO-2-METHYL-4-ITZ-3** | 5 | 0.25% | 1.42% |
| **PARABEN MIX 15%** | 4 | 0.20% | 1.14% |
| **THIOMERSAL 0.1%** | 3 | 0.15% | 0.85% |
| **MBT 2 %** | 3 | 0.15% | 0.85% |
| **PARATERTIARY BUTYL PHENOL 1%** | 3 | 0.15% | 0.85% |
| **WOOL ALCOHOLS 30%** | 3 | 0.15% | 0.85% |
| **BLACK RUBBER MIX 0.1%** | 3 | 0.15% | 0.85% |
| **PRIMIN 0.01 %** | 2 | 0.10% | 0.57% |
| **QUATERNIUM 15 1%** | 2 | 0.10% | 0.57% |
| **QUINOLINE MIX 6%** | 2 | 0.10% | 0.57% |
| **BENZOCAINE 5%** | 2 | 0.10% | 0.57% |
| **BENZALKONIUM CHLORIDE 0.1 %** | 1 | 0.05% | 0.28% |
| **MERCURY 0.05%** | 1 | 0.05% | 0.28% |
| **EPOXY RESIN 1%** | 1 | 0.05% | 0.28% |
| **MERCAPTO MIX 2%** | 1 | 0.05% | 0.28% |
| **PUPIL (WC, *n*=109** | | | |
|  | **Absolute *f(n)*** | ***f(%)* Total Population** | ***f(%)* Pupil Population** |
| **COBALT CHLORIDE 1%** | 14 | 0.71% | 12.84% |
| **NICKEL SULPHATE 5%** | 12 | 0.61% | 11.01% |
| **FRAGRANCE MIX (I) 8%** | 11 | 0.56% | 10.09% |
| **PARAPHENYLENEDIAMINE 1%** | 8 | 0.40% | 7.34% |
| **BALSAM OF PERU 25%** | 5 | 0.25% | 4.59% |
| **THIOMERSAL 0.1%** | 4 | 0.20% | 3.67% |
| **5-CHLORO-2-METHYL-4-ITZ-3** | 4 | 0.20% | 3.67% |
| **BLACK RUBBER MIX 0.1%** | 4 | 0.20% | 3.67% |
| **POTASSIUM DICHROMATE 0.5%** | 4 | 0.20% | 3.67% |
| **PARATERTIARY BUTYL PHENOL 1%** | 3 | 0.15% | 2.75% |
| **COLOPHONY 20%** | 3 | 0.15% | 2.75% |
| **NEOMYCIN SULPHATE 20%** | 3 | 0.15% | 2.75% |
| **PRIMIN 0.01 %** | 2 | 0.10% | 1.83% |
| **PARABEN MIX 15%** | 2 | 0.10% | 1.83% |
| **BUDESONIDE 0.01%** | 1 | 0.05% | 0.92% |
| **MERCURY 0.05%** | 1 | 0.05% | 0.92% |
| **QUATERNIUM 15 1%** | 1 | 0.05% | 0.92% |
| **ETHYLENEDIAMINE 1%** | 1 | 0.05% | 0.92% |
| **MERCAPTO MIX 2%** | 1 | 0.05% | 0.92% |
| **WOOL ALCOHOLS 30%** | 1 | 0.05% | 0.92% |
| **FORMALDEHYDE 2%** | 1 | 0.05% | 0.92% |
| **BENZOCAINE 5%** | 1 | 0.05% | 0.92% |
| **COSMETICS (BC, *n*=121)** | | | |
|  | **Absolute *f(n)*** | ***f(%)* Total Population** | ***f(%)* Cosmetics Population** |
| **NICKEL SULPHATE 5%** | 28 | 1.42% | 23.14% |
| **FRAGRANCE MIX (I) 8%** | 12 | 0.61% | 9.92% |
| **PARAPHENYLENEDIAMINE 1%** | 11 | 0.56% | 9.09% |
| **THIOMERSAL 0.1%** | 5 | 0.25% | 4.13% |
| **BALSAM OF PERU 25%** | 4 | 0.20% | 3.31% |
| **COBALT CHLORIDE 1%** | 4 | 0.20% | 3.31% |
| **QUATERNIUM 15 1%** | 3 | 0.15% | 2.48% |
| **THIURAM MIX 1%** | 3 | 0.15% | 2.48% |
| **POTASSIUM DICHROMATE 0.5%** | 3 | 0.15% | 2.48% |
| **BUDESONIDE 0.01%** | 2 | 0.10% | 1.65% |
| **BENZALKONIUM CHLORIDE 0.1 %** | 1 | 0.05% | 0.83% |
| **PRIMIN 0.01 %** | 1 | 0.05% | 0.83% |
| **5-CHLORO-2-METHYL-4-ITZ-3** | 1 | 0.05% | 0.83% |
| **ETHYLENEDIAMINE 1%** | 1 | 0.05% | 0.83% |
| **COLOPHONY 20%** | 1 | 0.05% | 0.83% |
| **FORMALDEHYDE 2%** | 1 | 0.05% | 0.83% |
| **NEOMYCIN SULPHATE 20%** | 1 | 0.05% | 0.83% |
| **BLUE COLLAR (BC, *n*=159)** | | | |
|  | **Absolute *f(n)*** | ***f(%)* Total Population** | ***f(%)* Blue Collar Population** |
| **NICKEL SULPHATE 5%** | 28 | 1.42% | 17.61% |
| **POTASSIUM DICHROMATE 0.5%** | 24 | 1.21% | 15.09% |
| **FRAGRANCE MIX (I) 8%** | 19 | 0.96% | 11.95% |
| **COBALT CHLORIDE 1%** | 19 | 0.96% | 11.95% |
| **BALSAM OF PERU 25%** | 17 | 0.86% | 10.69% |
| **THIOMERSAL 0.1%** | 13 | 0.66% | 8.18% |
| **THIURAM MIX 1%** | 13 | 0.66% | 8.18% |
| **ETHYLENEDIAMINE 1%** | 8 | 0.40% | 5.03% |
| **PARAPHENYLENEDIAMINE 1%** | 7 | 0.35% | 4.40% |
| **BUDESONIDE 0.01%** | 6 | 0.30% | 3.77% |
| **EPOXY RESIN 1%** | 6 | 0.30% | 3.77% |
| **WOOL ALCOHOLS 30%** | 5 | 0.25% | 3.14% |
| **COLOPHONY 20%** | 5 | 0.25% | 3.14% |
| **FORMALDEHYDE 2%** | 5 | 0.25% | 3.14% |
| **5-CHLORO-2-METHYL-4-ITZ-3** | 4 | 0.20% | 2.52% |
| **MERCAPTO MIX 2%** | 4 | 0.20% | 2.52% |
| **BENZOCAINE 5%** | 4 | 0.20% | 2.52% |
| **MERCURY 0.05%** | 3 | 0.15% | 1.89% |
| **NEOMYCIN SULPHATE 20%** | 3 | 0.15% | 1.89% |
| **PRIMIN 0.01 %** | 1 | 0.05% | 0.63% |
| **QUATERNIUM 15 1%** | 1 | 0.05% | 0.63% |
| **PARATERTIARY BUTYL PHENOL 1%** | 1 | 0.05% | 0.63% |
| **PARABEN MIX 15%** | 1 | 0.05% | 0.63% |
| **BLACK RUBBER MIX 0.1%** | 1 | 0.05% | 0.63% |
| **DRIVER (WC, *n*=24)** | | | |
|  | **Absolute *f(n)*** | ***f(%)* Total Population** | ***f(%)* Driver Population** |
| **NICKEL SULPHATE 5%** | 1 | 0.05% | 4.17% |
| **ETHYLENEDIAMINE 1%** | 1 | 0.05% | 4.17% |
| **BLACK RUBBER MIX 0.1%** | 1 | 0.05% | 4.17% |
| **BALSAM OF PERU 25%** | 1 | 0.05% | 4.17% |
| **NEOMYCIN SULPHATE 20%** | 1 | 0.05% | 4.17% |
| **DOMESTIC (BC, *n*=239)** | | | |
|  | **Absolute *f(n)*** | ***f(%)* Total Population** | ***f(%)* Domestic Population** |
| **NICKEL SULPHATE 5%** | 65 | 3.29% | 27.20% |
| **FRAGRANCE MIX (I) 8%** | 40 | 2.02% | 16.74% |
| **BALSAM OF PERU 25%** | 20 | 1.01% | 8.37% |
| **PARAPHENYLENEDIAMINE 1%** | 18 | 0.91% | 7.53% |
| **ETHYLENEDIAMINE 1%** | 12 | 0.61% | 5.02% |
| **COBALT CHLORIDE 1%** | 11 | 0.56% | 4.60% |
| **POTASSIUM DICHROMATE 0.5%** | 10 | 0.51% | 4.18% |
| **BUDESONIDE 0.01%** | 8 | 0.40% | 3.35% |
| **NEOMYCIN SULPHATE 20%** | 8 | 0.40% | 3.35% |
| **THIOMERSAL 0.1%** | 6 | 0.30% | 2.51% |
| **COLOPHONY 20%** | 6 | 0.30% | 2.51% |
| **THIURAM MIX 1%** | 6 | 0.30% | 2.51% |
| **WOOL ALCOHOLS 30%** | 5 | 0.25% | 2.09% |
| **BENZOCAINE 5%** | 4 | 0.20% | 1.67% |
| **PARATERTIARY BUTYL PHENOL 1%** | 3 | 0.15% | 1.26% |
| **QUINOLINE MIX 6%** | 3 | 0.15% | 1.26% |
| **FORMALDEHYDE 2%** | 3 | 0.15% | 1.26% |
| **5-CHLORO-2-METHYL-4-ITZ-3** | 2 | 0.10% | 0.84% |
| **PARABEN MIX 15%** | 2 | 0.10% | 0.84% |
| **EPOXY RESIN 1%** | 2 | 0.10% | 0.84% |
| **BLACK RUBBER MIX 0.1%** | 2 | 0.10% | 0.84% |
| **MERCURY 0.05%** | 1 | 0.05% | 0.42% |
| **QUATERNIUM 15 1%** | 1 | 0.05% | 0.42% |
| **MERCAPTO MIX 2%** | 1 | 0.05% | 0.42% |
| **SECURITY OFFICER (WC, *n*=13)** | | | |
|  | **Absolute *f(n)*** | ***f(%)* Total Population** | ***f(%)* Security Officer Population** |
| **NICKEL SULPHATE 5%** | 4 | 0.20% | 30.77% |
| **ETHYLENEDIAMINE 1%** | 2 | 0.10% | 15.38% |
| **FRAGRANCE MIX (I) 8%** | 2 | 0.10% | 15.38% |
| **PARAPHENYLENEDIAMINE 1%** | 2 | 0.10% | 15.38% |
| **THIOMERSAL 0.1%** | 1 | 0.05% | 7.69% |
| **5-CHLORO-2-METHYL-4-ITZ-3** | 1 | 0.05% | 7.69% |
| **QUATERNIUM 15 1%** | 1 | 0.05% | 7.69% |
| **PARATERTIARY BUTYL PHENOL 1%** | 1 | 0.05% | 7.69% |
| **BLACK RUBBER MIX 0.1%** | 1 | 0.05% | 7.69% |
| **BALSAM OF PERU 25%** | 1 | 0.05% | 7.69% |
| **COLOPHONY 20%** | 1 | 0.05% | 7.69% |
| **FORMALDEHYDE 2%** | 1 | 0.05% | 7.69% |
| **COBALT CHLORIDE 1%** | 1 | 0.05% | 7.69% |
| **ATHLETE (WC, *n*=12)** | | | |
|  | **Absolute *f(n)*** | ***f(%)* Total Population** | ***f(%)* Athlete Population** |
| **NICKEL SULPHATE 5%** | 3 | 0.15% | 25.00% |
| **BALSAM OF PERU 25%** | 2 | 0.10% | 16.67% |
| **COBALT CHLORIDE 1%** | 2 | 0.10% | 16.67% |
| **POTASSIUM DICHROMATE 0.5%** | 2 | 0.10% | 16.67% |
| **FRAGRANCE MIX (I) 8%** | 1 | 0.05% | 8.33% |
| **PARABEN MIX 15%** | 1 | 0.05% | 8.33% |
| **BLACK RUBBER MIX 0.1%** | 1 | 0.05% | 8.33% |
| **BENZOCAINE 5%** | 1 | 0.05% | 8.33% |
| **PARAPHENYLENEDIAMINE 1%** | 1 | 0.05% | 8.33% |
| **STUDENT (WC, *n*=86)** | | | |
|  | **Absolute *f(n)*** | ***f(%)* Total Population** | ***f(%)* Student Population** |
| **NICKEL SULPHATE 5%** | 20 | 1.01% | 23.26% |
| **THIOMERSAL 0.1%** | 15 | 0.76% | 17.44% |
| **POTASSIUM DICHROMATE 0.5%** | 6 | 0.30% | 6.98% |
| **COBALT CHLORIDE 1%** | 5 | 0.25% | 5.81% |
| **ETHYLENEDIAMINE 1%** | 3 | 0.15% | 3.49% |
| **FRAGRANCE MIX (I) 8%** | 3 | 0.15% | 3.49% |
| **FORMALDEHYDE 2%** | 3 | 0.15% | 3.49% |
| **PARAPHENYLENEDIAMINE 1%** | 3 | 0.15% | 3.49% |
| **QUATERNIUM 15 1%** | 2 | 0.10% | 2.33% |
| **BALSAM OF PERU 25%** | 2 | 0.10% | 2.33% |
| **NEOMYCIN SULPHATE 20%** | 2 | 0.10% | 2.33% |
| **PRIMIN 0.01 %** | 1 | 0.05% | 1.16% |
| **MBT 2 %** | 1 | 0.05% | 1.16% |
| **PARATERTIARY BUTYL PHENOL 1%** | 1 | 0.05% | 1.16% |
| **PARABEN MIX 15%** | 1 | 0.05% | 1.16% |
| **MERCAPTO MIX 2%** | 1 | 0.05% | 1.16% |
| **WOOL ALCOHOLS 30%** | 1 | 0.05% | 1.16% |
| **CLERK (WC, *n*=243)** | | | |
|  | **Absolute *f(n)*** | ***f(%)* Total Population** | ***f(%)* Clerk Population** |
| **NICKEL SULPHATE 5%** | 94 | 4.75% | 38.68% |
| **FRAGRANCE MIX (I) 8%** | 40 | 2.02% | 16.46% |
| **THIOMERSAL 0.1%** | 30 | 1.52% | 12.35% |
| **COBALT CHLORIDE 1%** | 29 | 1.47% | 11.93% |
| **BALSAM OF PERU 25%** | 20 | 1.01% | 8.23% |
| **POTASSIUM DICHROMATE 0.5%** | 14 | 0.71% | 5.76% |
| **PARAPHENYLENEDIAMINE 1%** | 13 | 0.66% | 5.35% |
| **BUDESONIDE 0.01%** | 9 | 0.46% | 3.70% |
| **ETHYLENEDIAMINE 1%** | 9 | 0.46% | 3.70% |
| **5-CHLORO-2-METHYL-4-ITZ-3** | 8 | 0.40% | 3.29% |
| **WOOL ALCOHOLS 30%** | 8 | 0.40% | 3.29% |
| **FORMALDEHYDE 2%** | 8 | 0.40% | 3.29% |
| **NEOMYCIN SULPHATE 20%** | 7 | 0.35% | 2.88% |
| **COLOPHONY 20%** | 6 | 0.30% | 2.47% |
| **PARATERTIARY BUTYL PHENOL 1%** | 4 | 0.20% | 1.65% |
| **THIURAM MIX 1%** | 4 | 0.20% | 1.65% |
| **BENZALKONIUM CHLORIDE 0.1 %** | 3 | 0.15% | 1.23% |
| **MERCURY 0.05%** | 3 | 0.15% | 1.23% |
| **BENZOCAINE 5%** | 3 | 0.15% | 1.23% |
| **PRIMIN 0.01 %** | 2 | 0.10% | 0.82% |
| **PARABEN MIX 15%** | 2 | 0.10% | 0.82% |
| **MERCAPTO MIX 2%** | 2 | 0.10% | 0.82% |
| **BLACK RUBBER MIX 0.1%** | 2 | 0.10% | 0.82% |
| **EPOXY RESIN 1%** | 1 | 0.05% | 0.41% |
| **PSYCHOLOGY (WC, *n*=8)** | | | |
|  | **Absolute *f(n)*** | ***f(%)* Total Population** | ***f(%)* Psychology Population** |
| **NICKEL SULPHATE 5%** | 5 | 0.25% | 62.50% |
| **FRAGRANCE MIX (I) 8%** | 3 | 0.15% | 37.50% |
| **ETHYLENEDIAMINE 1%** | 2 | 0.10% | 25.00% |
| **BLACK RUBBER MIX 0.1%** | 1 | 0.05% | 12.50% |
| **BALSAM OF PERU 25%** | 1 | 0.05% | 12.50% |
| **COLOPHONY 20%** | 1 | 0.05% | 12.50% |
| **FORMALDEHYDE 2%** | 1 | 0.05% | 12.50% |
| **COBALT CHLORIDE 1%** | 1 | 0.05% | 12.50% |
| **NEOMYCIN SULPHATE 20%** | 1 | 0.05% | 12.50% |
| **POTASSIUM DICHROMATE 0.5%** | 1 | 0.05% | 12.50% |
| **TEACHER (WC, *n*=78)** | | | |
|  | **Absolute *f(n)*** | ***f(%)* Total Population** | ***f(%)* Teacher Population** |
| **NICKEL SULPHATE 5%** | 34 | 1.72% | 43.59% |
| **FRAGRANCE MIX (I) 8%** | 17 | 0.86% | 21.79% |
| **THIOMERSAL 0.1%** | 11 | 0.56% | 14.10% |
| **BALSAM OF PERU 25%** | 8 | 0.40% | 10.26% |
| **5-CHLORO-2-METHYL-4-ITZ-3** | 7 | 0.35% | 8.97% |
| **COBALT CHLORIDE 1%** | 7 | 0.35% | 8.97% |
| **POTASSIUM DICHROMATE 0.5%** | 7 | 0.35% | 8.97% |
| **PARAPHENYLENEDIAMINE 1%** | 6 | 0.30% | 7.69% |
| **ETHYLENEDIAMINE 1%** | 5 | 0.25% | 6.41% |
| **FORMALDEHYDE 2%** | 5 | 0.25% | 6.41% |
| **NEOMYCIN SULPHATE 20%** | 4 | 0.20% | 5.13% |
| **BUDESONIDE 0.01%** | 2 | 0.10% | 2.56% |
| **QUATERNIUM 15 1%** | 2 | 0.10% | 2.56% |
| **PARATERTIARY BUTYL PHENOL 1%** | 2 | 0.10% | 2.56% |
| **PARABEN MIX 15%** | 2 | 0.10% | 2.56% |
| **QUINOLINE MIX 6%** | 2 | 0.10% | 2.56% |
| **BENZOCAINE 5%** | 2 | 0.10% | 2.56% |
| **THIURAM MIX 1%** | 2 | 0.10% | 2.56% |
| **PRIMIN 0.01 %** | 1 | 0.05% | 1.28% |
| **MERCAPTO MIX 2%** | 1 | 0.05% | 1.28% |
| **WOOL ALCOHOLS 30%** | 1 | 0.05% | 1.28% |
| **BLACK RUBBER MIX 0.1%** | 1 | 0.05% | 1.28% |
| **ATTORNEY (WC, *n*=23)** | | | |
|  | **Absolute *f(n)*** | ***f(%)* Total Population** | ***f(%)* Attorney Population** |
| **NICKEL SULPHATE 5%** | 9 | 0.46% | 39.13% |
| **FRAGRANCE MIX (I) 8%** | 5 | 0.25% | 21.74% |
| **THIOMERSAL 0.1%** | 2 | 0.10% | 8.70% |
| **BUDESONIDE 0.01%** | 1 | 0.05% | 4.35% |
| **ETHYLENEDIAMINE 1%** | 1 | 0.05% | 4.35% |
| **PARATERTIARY BUTYL PHENOL 1%** | 1 | 0.05% | 4.35% |
| **COBALT CHLORIDE 1%** | 1 | 0.05% | 4.35% |
| **NEOMYCIN SULPHATE 20%** | 1 | 0.05% | 4.35% |
| **ARTIST (WC, *n*=29)** | | | |
|  | **Absolute *f(n)*** | ***f(%)* Total Population** | ***f(%)* Artist Population** |
| **NICKEL SULPHATE 5%** | 11 | 0.56% | 37.93% |
| **FRAGRANCE MIX (I) 8%** | 5 | 0.25% | 17.24% |
| **THIOMERSAL 0.1%** | 4 | 0.20% | 13.79% |
| **5-CHLORO-2-METHYL-4-ITZ-3** | 2 | 0.10% | 6.90% |
| **PARATERTIARY BUTYL PHENOL 1%** | 2 | 0.10% | 6.90% |
| **BALSAM OF PERU 25%** | 2 | 0.10% | 6.90% |
| **COBALT CHLORIDE 1%** | 2 | 0.10% | 6.90% |
| **PARAPHENYLENEDIAMINE 1%** | 2 | 0.10% | 6.90% |
| **POTASSIUM DICHROMATE 0.5%** | 2 | 0.10% | 6.90% |
| **WOOL ALCOHOLS 30%** | 1 | 0.05% | 3.45% |
| **COLOPHONY 20%** | 1 | 0.05% | 3.45% |
| **NEOMYCIN SULPHATE 20%** | 1 | 0.05% | 3.45% |
| **PUBLIC SERVANTS (WC, *n*=33)** | | | |
|  | **Absolute *f(n)*** | ***f(%)* Total Population** | ***f(%)* Public Servants Population** |
| **NICKEL SULPHATE 5%** | 12 | 0.61% | 36.36% |
| **FRAGRANCE MIX (I) 8%** | 6 | 0.30% | 18.18% |
| **COBALT CHLORIDE 1%** | 6 | 0.30% | 18.18% |
| **THIOMERSAL 0.1%** | 4 | 0.20% | 12.12% |
| **5-CHLORO-2-METHYL-4-ITZ-3** | 3 | 0.15% | 9.09% |
| **FORMALDEHYDE 2%** | 3 | 0.15% | 9.09% |
| **BUDESONIDE 0.01%** | 2 | 0.10% | 6.06% |
| **QUATERNIUM 15 1%** | 2 | 0.10% | 6.06% |
| **BALSAM OF PERU 25%** | 2 | 0.10% | 6.06% |
| **ETHYLENEDIAMINE 1%** | 1 | 0.05% | 3.03% |
| **EPOXY RESIN 1%** | 1 | 0.05% | 3.03% |
| **QUINOLINE MIX 6%** | 1 | 0.05% | 3.03% |
| **NEOMYCIN SULPHATE 20%** | 1 | 0.05% | 3.03% |
| **POTASSIUM DICHROMATE 0.5%** | 1 | 0.05% | 3.03% |
| **DOMESTICS (WC, *n*=38)** | | | |
|  | **Absolute *f(n)*** | ***f(%)* Total Population** | ***f(%)* Domestics Population** |
| **NICKEL SULPHATE 5%** | 13 | 0.66% | 34.21% |
| **FRAGRANCE MIX (I) 8%** | 7 | 0.35% | 18.42% |
| **ETHYLENEDIAMINE 1%** | 4 | 0.20% | 10.53% |
| **BALSAM OF PERU 25%** | 4 | 0.20% | 10.53% |
| **COBALT CHLORIDE 1%** | 4 | 0.20% | 10.53% |
| **COLOPHONY 20%** | 3 | 0.15% | 7.89% |
| **POTASSIUM DICHROMATE 0.5%** | 3 | 0.15% | 7.89% |
| **5-CHLORO-2-METHYL-4-ITZ-3** | 2 | 0.10% | 5.26% |
| **QUATERNIUM 15 1%** | 2 | 0.10% | 5.26% |
| **PARATERTIARY BUTYL PHENOL 1%** | 2 | 0.10% | 5.26% |
| **FORMALDEHYDE 2%** | 2 | 0.10% | 5.26% |
| **THIURAM MIX 1%** | 2 | 0.10% | 5.26% |
| **THIOMERSAL 0.1%** | 1 | 0.05% | 2.63% |
| **BUDESONIDE 0.01%** | 1 | 0.05% | 2.63% |
| **PRIMIN 0.01 %** | 1 | 0.05% | 2.63% |
| **MERCURY 0.05%** | 1 | 0.05% | 2.63% |
| **MERCAPTO MIX 2%** | 1 | 0.05% | 2.63% |
| **PARAPHENYLENEDIAMINE 1%** | 1 | 0.05% | 2.63% |
| **ARMY OFFICER (WC, *n*=4)** | | | |
|  | **Absolute *f(n)*** | ***f(%)* Total Population** | ***f(%)* Army Officer Population** |
| **THIOMERSAL 0.1%** | 2 | 0.10% | 50.00% |
| **BUDESONIDE 0.01%** | 1 | 0.05% | 25.00% |
| **NICKEL SULPHATE 5%** | 1 | 0.05% | 25.00% |
| **BALSAM OF PERU 25%** | 1 | 0.05% | 25.00% |
| **SOCIOLOGY (WC, *n*=7)** | | | |
|  | **Absolute *f(n)*** | ***f(%)* Total Population** | ***f(%)* Sociology Population** |
| **NICKEL SULPHATE 5%** | 3 | 0.15% | 42.86% |
| **THIOMERSAL 0.1%** | 1 | 0.05% | 14.29% |
| **QUATERNIUM 15 1%** | 1 | 0.05% | 14.29% |
| **FRAGRANCE MIX (I) 8%** | 1 | 0.05% | 14.29% |
| **PARATERTIARY BUTYL PHENOL 1%** | 1 | 0.05% | 14.29% |
| **BALSAM OF PERU 25%** | 1 | 0.05% | 14.29% |
| **FORMALDEHYDE 2%** | 1 | 0.05% | 14.29% |
| **COBALT CHLORIDE 1%** | 1 | 0.05% | 14.29% |
| **NEOMYCIN SULPHATE 20%** | 1 | 0.05% | 14.29% |
| **UNEMPLOYED (WC, *n*=89)** | | | |
|  | **Absolute *f(n)*** | ***f(%)* Total Population** | ***f(%)* Unemployed Population** |
| **NICKEL SULPHATE 5%** | 25 | 1.26% | 28.09% |
| **FRAGRANCE MIX (I) 8%** | 14 | 0.71% | 15.73% |
| **BALSAM OF PERU 25%** | 11 | 0.56% | 12.36% |
| **COBALT CHLORIDE 1%** | 8 | 0.40% | 8.99% |
| **ETHYLENEDIAMINE 1%** | 7 | 0.35% | 7.87% |
| **THIOMERSAL 0.1%** | 5 | 0.25% | 5.62% |
| **PARAPHENYLENEDIAMINE 1%** | 5 | 0.25% | 5.62% |
| **WOOL ALCOHOLS 30%** | 3 | 0.15% | 3.37% |
| **POTASSIUM DICHROMATE 0.5%** | 3 | 0.15% | 3.37% |
| **PRIMIN 0.01 %** | 2 | 0.10% | 2.25% |
| **5-CHLORO-2-METHYL-4-ITZ-3** | 2 | 0.10% | 2.25% |
| **BLACK RUBBER MIX 0.1%** | 2 | 0.10% | 2.25% |
| **THIURAM MIX 1%** | 2 | 0.10% | 2.25% |
| **NEOMYCIN SULPHATE 20%** | 2 | 0.10% | 2.25% |
| **BUDESONIDE 0.01%** | 1 | 0.05% | 1.12% |
| **QUATERNIUM 15 1%** | 1 | 0.05% | 1.12% |
| **PARABEN MIX 15%** | 1 | 0.05% | 1.12% |
| **EPOXY RESIN 1%** | 1 | 0.05% | 1.12% |
| **COLOPHONY 20%** | 1 | 0.05% | 1.12% |
| **FORMALDEHYDE 2%** | 1 | 0.05% | 1.12% |
| **BENZOCAINE 5%** | 1 | 0.05% | 1.12% |
| **JOURNALIST (WC, *n*=5)** | | | |
|  | **Absolute *f(n)*** | ***f(%)* Total Population** | ***f(%)* Journalist Population** |
| **5-CHLORO-2-METHYL-4-ITZ-3** | 2 | 0.10% | 40.00% |
| **NICKEL SULPHATE 5%** | 1 | 0.05% | 20.00% |
| **ETHYLENEDIAMINE 1%** | 1 | 0.05% | 20.00% |
| **DENTIST (WC, *n*=6)** | | | |
|  | **Absolute *f(n)*** | ***f(%)* Total Population** | ***f(%)* Dentist Population** |
| **THIOMERSAL 0.1%** | 2 | 0.10% | 33.33% |
| **FRAGRANCE MIX (I) 8%** | 2 | 0.10% | 33.33% |
| **NICKEL SULPHATE 5%** | 1 | 0.05% | 16.67% |
| **ETHYLENEDIAMINE 1%** | 1 | 0.05% | 16.67% |
| **PHYSICIAN (WC, *n*=18)** | | | |
|  | **Absolute *f(n)*** | ***f(%)* Total Population** | ***f(%)* Physician Population** |
| **NICKEL SULPHATE 5%** | 6 | 0.30% | 33.33% |
| **THIOMERSAL 0.1%** | 3 | 0.15% | 16.67% |
| **COBALT CHLORIDE 1%** | 2 | 0.10% | 11.11% |
| **FRAGRANCE MIX (I) 8%** | 1 | 0.05% | 5.56% |
| **FORMALDEHYDE 2%** | 1 | 0.05% | 5.56% |
| **THIURAM MIX 1%** | 1 | 0.05% | 5.56% |
| **PARAMEDICAL (WC, *n*=25)** | | | |
|  | **Absolute *f(n)*** | ***f(%)* Total Population** | ***f(%)* Paramedical Population** |
| **NICKEL SULPHATE 5%** | 12 | 0.61% | 48.00% |
| **COBALT CHLORIDE 1%** | 4 | 0.20% | 16.00% |
| **FRAGRANCE MIX (I) 8%** | 3 | 0.15% | 12.00% |
| **THIOMERSAL 0.1%** | 2 | 0.10% | 8.00% |
| **PARABEN MIX 15%** | 2 | 0.10% | 8.00% |
| **BALSAM OF PERU 25%** | 2 | 0.10% | 8.00% |
| **FORMALDEHYDE 2%** | 2 | 0.10% | 8.00% |
| **POTASSIUM DICHROMATE 0.5%** | 2 | 0.10% | 8.00% |
| **BUDESONIDE 0.01%** | 1 | 0.05% | 4.00% |
| **MERCURY 0.05%** | 1 | 0.05% | 4.00% |
| **5-CHLORO-2-METHYL-4-ITZ-3** | 1 | 0.05% | 4.00% |
| **MERCAPTO MIX 2%** | 1 | 0.05% | 4.00% |
| **BLACK RUBBER MIX 0.1%** | 1 | 0.05% | 4.00% |
| **PARAPHENYLENEDIAMINE 1%** | 1 | 0.05% | 4.00% |
| **CHILD (WC, *n*=6)** | | | |
|  | **Absolute *f(n)*** | ***f(%)* Total Population** | ***f(%)* Children Population** |
| **NICKEL SULPHATE 5%** | 1 | 0.05% | 16.67% |
| **PARABEN MIX 15%** | 1 | 0.05% | 16.67% |
| **COOK (BC, *n*=15)** | | | |
|  | **Absolute *f(n)*** | ***f(%)* Total Population** | ***f(%)* Cook Population** |
| **ETHYLENEDIAMINE 1%** | 3 | 0.15% | 20.00% |
| **NICKEL SULPHATE 5%** | 2 | 0.10% | 13.33% |
| **COBALT CHLORIDE 1%** | 2 | 0.10% | 13.33% |
| **BUDESONIDE 0.01%** | 1 | 0.05% | 6.67% |
| **THIURAM MIX 1%** | 1 | 0.05% | 6.67% |
| **POTASSIUM DICHROMATE 0.5%** | 1 | 0.05% | 6.67% |
| **NURSE (WC, *n*=36)** | | | |
|  | **Absolute *f(n)*** | ***f(%)* Total Population** | ***f(%)* Nurse Population** |
| **NICKEL SULPHATE 5%** | 15 | 0.76% | 41.67% |
| **THIOMERSAL 0.1%** | 6 | 0.30% | 16.67% |
| **FRAGRANCE MIX (I) 8%** | 4 | 0.20% | 11.11% |
| **BLACK RUBBER MIX 0.1%** | 3 | 0.15% | 8.33% |
| **COBALT CHLORIDE 1%** | 3 | 0.15% | 8.33% |
| **ETHYLENEDIAMINE 1%** | 2 | 0.10% | 5.56% |
| **MERCAPTO MIX 2%** | 2 | 0.10% | 5.56% |
| **BALSAM OF PERU 25%** | 2 | 0.10% | 5.56% |
| **PARAPHENYLENEDIAMINE 1%** | 2 | 0.10% | 5.56% |
| **POTASSIUM DICHROMATE 0.5%** | 2 | 0.10% | 5.56% |
| **MERCURY 0.05%** | 1 | 0.05% | 2.78% |
| **5-CHLORO-2-METHYL-4-ITZ-3** | 1 | 0.05% | 2.78% |
| **WOOL ALCOHOLS 30%** | 1 | 0.05% | 2.78% |
| **QUINOLINE MIX 6%** | 1 | 0.05% | 2.78% |
| **BENZOCAINE 5%** | 1 | 0.05% | 2.78% |
| **THIURAM MIX 1%** | 1 | 0.05% | 2.78% |
| **NEOMYCIN SULPHATE 20%** | 1 | 0.05% | 2.78% |
| **SCIENTIST (WC, *n*=16)** | | | |
|  | **Absolute *f(n)*** | ***f(%)* Total Population** | ***f(%)* Scientists Population** |
| **NICKEL SULPHATE 5%** | 2 | 0.10% | 12.50% |
| **ETHYLENEDIAMINE 1%** | 2 | 0.10% | 12.50% |
| **BALSAM OF PERU 25%** | 2 | 0.10% | 12.50% |
| **THIOMERSAL 0.1%** | 1 | 0.05% | 6.25% |
| **FRAGRANCE MIX (I) 8%** | 1 | 0.05% | 6.25% |
| **EPOXY RESIN 1%** | 1 | 0.05% | 6.25% |
| **BLACK RUBBER MIX 0.1%** | 1 | 0.05% | 6.25% |
| **COBALT CHLORIDE 1%** | 1 | 0.05% | 6.25% |
| **PARAPHENYLENEDIAMINE 1%** | 1 | 0.05% | 6.25% |
| **NEOMYCIN SULPHATE 20%** | 1 | 0.05% | 6.25% |
| **BUSINESSMAN (WC, *n*=43)** | | | |
|  | **Absolute *f(n)*** | ***f(%)* Total Population** | ***f(%)* Businessman Population** |
| **NICKEL SULPHATE 5%** | 14 | 0.71% | 32.56% |
| **FRAGRANCE MIX (I) 8%** | 5 | 0.25% | 11.63% |
| **ETHYLENEDIAMINE 1%** | 4 | 0.20% | 9.30% |
| **NEOMYCIN SULPHATE 20%** | 3 | 0.15% | 6.98% |
| **BALSAM OF PERU 25%** | 2 | 0.10% | 4.65% |
| **FORMALDEHYDE 2%** | 2 | 0.10% | 4.65% |
| **COBALT CHLORIDE 1%** | 2 | 0.10% | 4.65% |
| **THIOMERSAL 0.1%** | 1 | 0.05% | 2.33% |
| **5-CHLORO-2-METHYL-4-ITZ-3** | 1 | 0.05% | 2.33% |
| **QUATERNIUM 15 1%** | 1 | 0.05% | 2.33% |
| **PARABEN MIX 15%** | 1 | 0.05% | 2.33% |
| **QUINOLINE MIX 6%** | 1 | 0.05% | 2.33% |
| **COLOPHONY 20%** | 1 | 0.05% | 2.33% |
| **THIURAM MIX 1%** | 1 | 0.05% | 2.33% |
| **POTASSIUM DICHROMATE 0.5%** | 1 | 0.05% | 2.33% |
| **ENGINEER (WC, *n*=43)** | | | |
|  | **Absolute *f(n)*** | ***f(%)* Total Population** | ***f(%)* Engineers Population** |
| **NICKEL SULPHATE 5%** | 13 | 0.66% | 30.23% |
| **FRAGRANCE MIX (I) 8%** | 7 | 0.35% | 16.28% |
| **THIOMERSAL 0.1%** | 6 | 0.30% | 13.95% |
| **BALSAM OF PERU 25%** | 5 | 0.25% | 11.63% |
| **ETHYLENEDIAMINE 1%** | 4 | 0.20% | 9.30% |
| **COBALT CHLORIDE 1%** | 4 | 0.20% | 9.30% |
| **PARAPHENYLENEDIAMINE 1%** | 4 | 0.20% | 9.30% |
| **THIURAM MIX 1%** | 3 | 0.15% | 6.98% |
| **NEOMYCIN SULPHATE 20%** | 3 | 0.15% | 6.98% |
| **BUDESONIDE 0.01%** | 2 | 0.10% | 4.65% |
| **5-CHLORO-2-METHYL-4-ITZ-3** | 2 | 0.10% | 4.65% |
| **BENZALKONIUM CHLORIDE 0.1 %** | 1 | 0.05% | 2.33% |
| **PRIMIN 0.01 %** | 1 | 0.05% | 2.33% |
| **QUATERNIUM 15 1%** | 1 | 0.05% | 2.33% |
| **PARATERTIARY BUTYL PHENOL 1%** | 1 | 0.05% | 2.33% |
| **BLACK RUBBER MIX 0.1%** | 1 | 0.05% | 2.33% |
| **COLOPHONY 20%** | 1 | 0.05% | 2.33% |
| **BENZOCAINE 5%** | 1 | 0.05% | 2.33% |
| **POTASSIUM DICHROMATE 0.5%** | 1 | 0.05% | 2.33% |
| **AGRICULTURE (BC, *n*=27)** | | | |
|  | **Absolute *f(n)*** | ***f(%)* Total Population** | ***f(%)* Agriculture Population** |
| **NICKEL SULPHATE 5%** | 5 | 0.25% | 18.52% |
| **BALSAM OF PERU 25%** | 5 | 0.25% | 18.52% |
| **5-CHLORO-2-METHYL-4-ITZ-3** | 3 | 0.15% | 11.11% |
| **ETHYLENEDIAMINE 1%** | 3 | 0.15% | 11.11% |
| **FRAGRANCE MIX (I) 8%** | 2 | 0.10% | 7.41% |
| **POTASSIUM DICHROMATE 0.5%** | 2 | 0.10% | 7.41% |
| **BENZALKONIUM CHLORIDE 0.1 %** | 1 | 0.05% | 3.70% |
| **FORMALDEHYDE 2%** | 1 | 0.05% | 3.70% |
| **PARAPHENYLENEDIAMINE 1%** | 1 | 0.05% | 3.70% |
| **THIURAM MIX 1%** | 1 | 0.05% | 3.70% |
| **NEOMYCIN SULPHATE 20%** | 1 | 0.05% | 3.70% |
| **INFORMATICS (WC, *n*=6)** | | | |
|  | **Absolute *f(n)*** | ***f(%)* Total Population** | ***f(%)* Informatics Population** |
| **THIOMERSAL 0.1%** | 3 | 0.15% | 50.00% |
| **NICKEL SULPHATE 5%** | 2 | 0.10% | 33.33% |
| **BALSAM OF PERU 25%** | 1 | 0.05% | 16.67% |
| **WAITER (BC, *n*=15)** | | | |
|  | **Absolute *f(n)*** | ***f(%)* Total Population** | ***f(%)* Waiter Population** |
| **THIOMERSAL 0.1%** | 2 | 0.10% | 13.33% |
| **NICKEL SULPHATE 5%** | 2 | 0.10% | 13.33% |
| **FORMALDEHYDE 2%** | 2 | 0.10% | 13.33% |
| **PARAPHENYLENEDIAMINE 1%** | 2 | 0.10% | 13.33% |
| **5-CHLORO-2-METHYL-4-ITZ-3** | 1 | 0.05% | 6.67% |
| **BLACK RUBBER MIX 0.1%** | 1 | 0.05% | 6.67% |
| **BENZOCAINE 5%** | 1 | 0.05% | 6.67% |
| **PHARMACIST (WC, *n*=4)** | | | |
|  | **Absolute *f(n)*** | ***f(%)* Total Population** | ***f(%)* Pharmacist Population** |
| **NICKEL SULPHATE 5%** | 2 | 0.10% | 50.00% |
| **THIOMERSAL 0.1%** | 1 | 0.05% | 25.00% |
| **5-CHLORO-2-METHYL-4-ITZ-3** | 1 | 0.05% | 25.00% |
| **ETHYLENEDIAMINE 1%** | 1 | 0.05% | 25.00% |
| **FRAGRANCE MIX (I) 8%** | 1 | 0.05% | 25.00% |
| **PARABEN MIX 15%** | 1 | 0.05% | 25.00% |
| **BALSAM OF PERU 25%** | 1 | 0.05% | 25.00% |
| **COLOPHONY 20%** | 1 | 0.05% | 25.00% |
| **BENZOCAINE 5%** | 1 | 0.05% | 25.00% |
| **PARAPHENYLENEDIAMINE 1%** | 1 | 0.05% | 25.00% |
| **NAVY (BC, *n*=7)** | | | |
|  | **Absolute *f(n)*** | ***f(%)* Total Population** | ***f(%)* Navy Population** |
| **THIOMERSAL 0.1%** | 3 | 0.15% | 42.86% |
| **5-CHLORO-2-METHYL-4-ITZ-3** | 1 | 0.05% | 14.29% |
| **NICKEL SULPHATE 5%** | 1 | 0.05% | 14.29% |
| **COBALT CHLORIDE 1%** | 1 | 0.05% | 14.29% |
| **POTASSIUM DICHROMATE 0.5%** | 1 | 0.05% | 14.29% |
| **VETERINERIAN (WC, *n*=2)** | | | |
|  | **Absolute *f(n)*** | ***f(%)* Total Population** | ***f(%)* Veterinerian Population** |
| **NICKEL SULPHATE 5%** | 1 | 0.05% | 50.00% |
| **BALSAM OF PERU 25%** | 1 | 0.05% | 50.00% |
| **PARAPHENYLENEDIAMINE 1%** | 1 | 0.05% | 50.00% |
| **PROFESSOR (WC, *n*=4)** | | | |
|  | **Absolute *f(n)*** | ***f(%)* Total Population** | ***f(%)* Professor Population** |
| **5-CHLORO-2-METHYL-4-ITZ-3** | 1 | 0.05% | 25.00% |
| **NICKEL SULPHATE 5%** | 1 | 0.05% | 25.00% |
| **ETHYLENEDIAMINE 1%** | 1 | 0.05% | 25.00% |
| **BALSAM OF PERU 25%** | 1 | 0.05% | 25.00% |
| **OTHER (WC, *n*=3)** | | | |
|  | **Absolute *f(n)*** | ***f(%)* Total Population** | ***f(%)* Other Population** |
| **FRAGRANCE MIX (I) 8%** | 2 | 0.10% | 66.67% |
| **BALSAM OF PERU 25%** | 2 | 0.10% | 66.67% |
| **NICKEL SULPHATE 5%** | 1 | 0.05% | 33.33% |
| **MERCAPTO MIX 2%** | 1 | 0.05% | 33.33% |
| **BLACK RUBBER MIX 0.1%** | 1 | 0.05% | 33.33% |
| **FORMALDEHYDE 2%** | 1 | 0.05% | 33.33% |

**Supplementary Table 2**. Frequencies of the 28 allergens of the EBS. Frequencies are presented in descending order calculated for each detailed occupation.
